# Favorable winds speed up bird migration in spring but not in autumn

**DOI:** 10.1002/ece3.9146

**Published:** 2022-07-31

**Authors:** Raphaël Nussbaumer, Baptiste Schmid, Silke Bauer, Felix Liechti

**Affiliations:** ^1^ Cornell Lab of Ornithology Cornell University Ithaca New York USA; ^2^ Swiss Ornithological Institute Sempach Switzerland

**Keywords:** aeroecology, airspeed, ground speed, movement, season, weather radar

## Abstract

Wind has a significant yet complex effect on bird migration speed. With prevailing south wind, overall migration is generally faster in spring than in autumn. However, studies on the difference in airspeed between seasons have shown contrasting results so far, in part due to their limited geographical or temporal coverage. Using the first full‐year weather radar data set of nocturnal bird migration across western Europe together with wind speed from reanalysis data, we investigate variation of airspeed across season. We additionally expand our analysis of ground speed, airspeed, wind speed, and wind profit variation across time (seasonal and daily) and space (geographical and altitudinal). Our result confirms that wind plays a major role in explaining both temporal and spatial variabilities in ground speed. The resulting airspeed remains relatively constant at all scales (daily, seasonal, geographically and altitudinally). We found that spring airspeed is overall 5% faster in Spring than autumn, but we argue that this number is not significant compared to the biases and limitation of weather radar data. The results of the analysis can be used to further investigate birds' migratory strategies across space and time, as well as their energy use.

## Graphical abstract

Using the first full year of weather radar data in western Europe, we investigate the difference of airspeed accros time (daily and seasonally) and space (geographically and altitudinal). Our result show that wind plays a major role in explaining the variation of groundspeed, while airspeed remains constant through space and time.

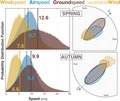

## 1 INTRODUCTION

Wind is a crucial parameter influencing bird migration, affecting bird survival and ultimately shaping migration routes (Erni et al., 2005; Kranstauber et al., 2015; la Sorte et al., 2014). This is because, with a similar order of magnitude as the birds' airspeed, favorable wind conditions can considerably increase the speed of migration, which reduces the energy required of birds to perform their migration journey and improves their survival (Alerstam & Lindström, 1990; Liechti, 2006; Richardson, 1978, 1990; Shamoun‐Baranes et al., 2017). The increase of ground speed brought by winds pays off on two fronts: birds can increase the distance covered for a given flight time, or reduce the energy cost for a given distance, thus shortening the time required for refueling.

Due to dominant global wind patterns and opposite directions of (return‐) migration, wind affects spring and autumn migration differently. Indeed, the stronger supporting winds in spring over autumn contribute to faster migration speed in spring (Gauthreaux et al., 2005; Kemp et al., 2010; la Sorte et al., 2014), in particular when birds can benefit from low‐level jet (Liechti & Schaller, 1999; Wainwright et al., 2016). This seasonal difference in average ground speed has been confirmed by multiple (radar) studies (Felix et al., 2008; Horton, van Doren, Stepanian, Farnsworth, & Kelly, 2016a; la Sorte et al., 2018; Nilsson et al., 2014), but see (Liechti & Bruderer, 1995). In addition, favorable winds occur more often in spring, giving birds more opportunities to initiate migratory bouts and reducing overall migration duration. This seasonal difference in prevalence of favorable winds, combined with the lower energy cost per distance described above, also explains the shorter stopovers in spring (Nilsson et al., 2013; Tøttrup et al., 2012).

Beyond the influence of wind increasing ground speed, it has been hypothesized that birds increase their airspeed in spring (Nilsson et al., 2014). Indeed, arriving at the breeding area before competitors has shown to improve reproductive output (e.g., Forstmeier, 2002; Gilsenan et al., 2020; Kokko, 1999; Reséndiz‐Infante & Gauthier, 2020) but arriving *too early* can hinder birds' survival due to lack of resources (Lerche‐Jørgensen et al., 2018). Assuming more competition at breeding than wintering site, birds would prioritize a shorter migration in spring and a lower energy expenditure in autumn, resulting in higher airspeeds during spring migration (Hedenstrom & Alerstam, 1995). However, an increase in airspeed comes with higher flight energy costs, which result in prolonged stopovers. According to optimal flight theory (e.g., Alerstam & Lindström, 1990), birds should fly slightly faster (5–15%) when minimizing their overall migration duration (including replenishment at stopover) rather than when minimizing the overall energy used (Alerstam, 2003; Nilsson et al., 2013). However, given that flight represents only 6.5% of migration time (Briedis et al., 2020), the corresponding time gained over the entire migration journey in prioritizing speed over energy is less than 1% (Hedenstrom & Alerstam, 1998). Thus, if the goal of spring migration is to arrive earlier, flying faster only has a limited impact and there is stronger selectivity on replenishment during stopovers than airspeed (Houston, 2000).

Compared to the overall migration speed which can be more readily estimated (e.g., Briedis et al., 2020; Fransson, 1995; la Sorte et al., 2013; Yohannes et al., 2009), airspeed is harder to measure. Weather radars are well positioned to do so, yet so far have shown mixed results: some showing significantly faster airspeed in spring (Henningsson et al., 2009; Horton, van Doren, Stepanian, Farnsworth, & Kelly, 2016a; Karlsson et al., 2012; Nilsson et al., 2014), others similar speeds in both seasons (Liechti & Bruderer, 1995) and yet others finding slightly faster airspeeds in autumn (Kemp et al., 2010).

To date, these earlier studies have been conducted with data sets that are either geographically limited or do not cover the entire year. We draw on the first full year data set of nocturnal migration captured by European weather radars and combine it with high resolution weather re‐analysis data to re‐assess the relative effect of wind speed (and orientation) on the birds' airspeed. To further compare the seasonal difference in ground speed and airspeed, we investigate intraseasonal, geographical, and altitudinal differences.

## 2 MATERIAL AND METHODS

### 2.1 Data

#### 2.1.1 Weather radar data: Bird vector speed and density

The vertical profile time series (Nussbaumer, 2020) consists of bird density ρ [bird/km^3^], ground speed along the east–west u and south–north v components [m/s], and radial velocity standard deviation (a measure of the directional scattering of the speed) extracted from 37 weather radars in western Europe using vol2bird (Dokter et al., 2011, 2019). The final data set consists of 6.8 million datapoints spanning from February 13, 2018 to January 1, 2019, with a temporal resolution of 5 min and spatial resolution of 200 m in altitude (0–5 km). Details on the preprocessing procedure are provided in Nussbaumer et al. (2021).

#### 2.1.2 Climate reanalysis: Wind vector speed at pressure level

The east–west U and south–north V components of wind speed were retrieved from the ERA5 reanalysis (Hersbach et al., 2018). We downloaded the data at the maximal resolution (hourly, 0.25°× 0.25° and pressure level from 1000 to 550 hPa) for the year 2018. Both components U and V were linearly interpolated (time–space 4D) at each datapoint of the weather radar data.

### 2.2 Analysis

We compare ground speed, airspeed, experienced wind speed, available wind speed, and wind profit in spring and autumn (taking 15 July as cut‐off day) at four different scales: (1) seasonal, (2) daily (i.e., within season), (3) geographical, and (4) altitudinal.

Using the triangle of velocities (e.g., Alerstam & Hedenstrom, 1998), bird ground speed ($V_{g}$) and airspeed ($V_{a}$) can be computed respectively with

$$V_{g}=\sqrt{u^{2}+v^{2}}$$

and

$$V_{a}=\sqrt{{\left(u-U\right)}^{2}+{\left(v-V\right)}^{2}}.$$

Thus, airspeed is computed locally for each datapoint accounting for the specific wind speed and orientation at this location.

In the analysis, we differentiate between experienced wind speed and available wind speed by using a weighted average based on bird density for the experienced wind speed and simple (unweighted) average for the available wind speed.

Finally, wind profit is computed as the vector projection of the wind speed on the assumed bird migration direction of 225° (e.g., Bruderer & Jenni, 1990), with a north‐east orientation in spring and south‐west in autumn.

## 3 RESULTS

### 3.1 Seasonal scale

**FIGURE 1:**
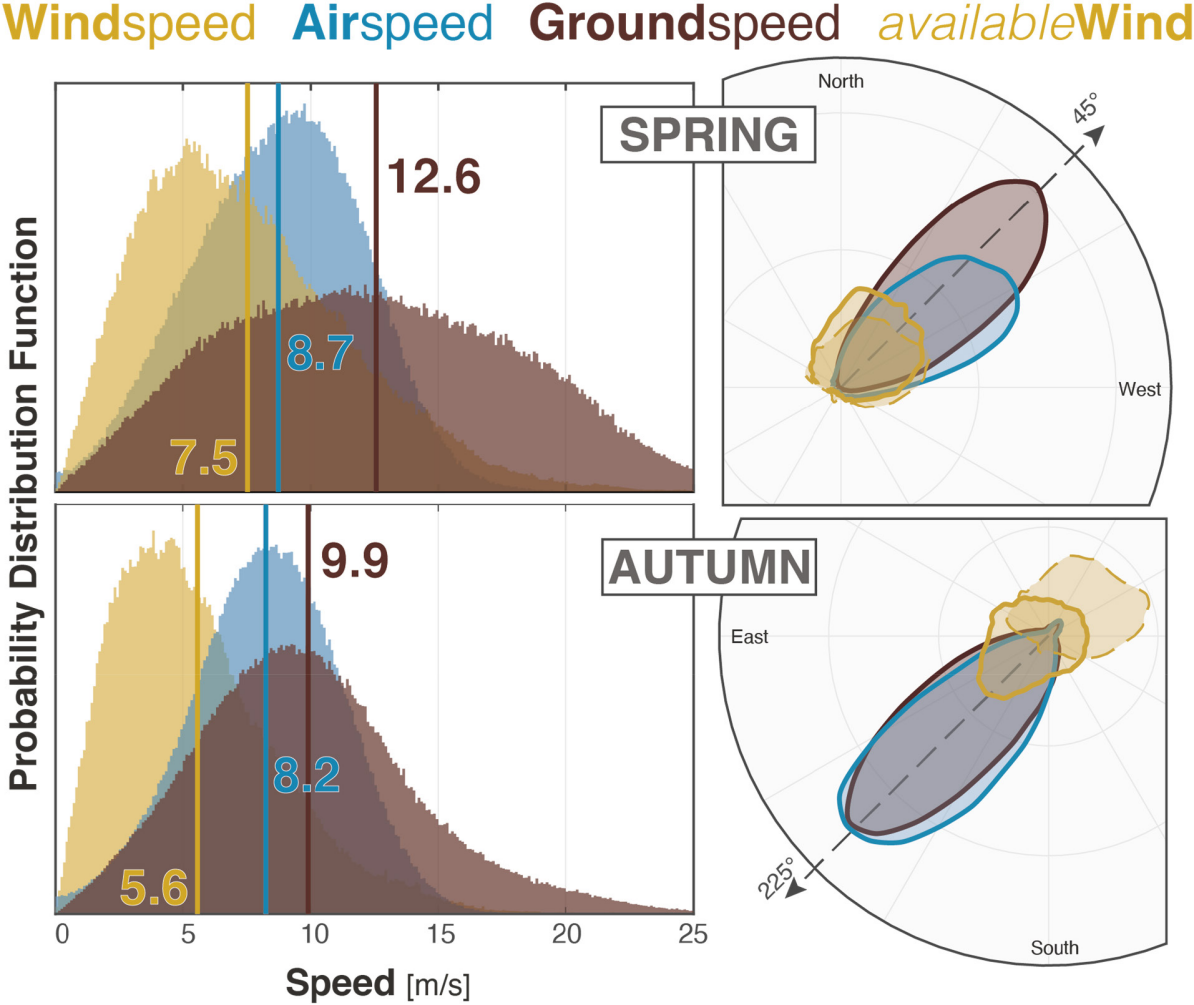
(Left) histogram of ground speed (brown), airspeed (blue), and wind speed (yellow) per season. The distribution of airspeed remains relatively similar between seasons, while wind speed and ground speed are greater in spring (vertical lines indicate the arithmetic means). (Right) Polar histogram of direction per season. The 45° and 225° dashed lines indicate the prefered directions of migration used for the calculation of windprofit. Wind speed direction is generally more spread, particularly in autumn. In autumn, birds are more selective of the wind oriented toward their prefered direction of migration all quantities (speed and directions) are weighted by the number of birds except for available winds (dashed histogram).

In general, the wind speed experienced by birds was higher in spring than in autumn (average windspeed of 7.5 vs 5.6 m/s, see Figure 1). The difference of wind speed was caused by the predominant Southwest winds, producing a higher wind profit in spring (avg of 4.6 m/s) than in autumn (1.1 m/s) (see Figure SI‐3). Most of this increase was also observed in the ground speed (12.6 vs 9.9 m/s), resulting in strikingly similar airspeeds between the two seasons (8.7 vs 8.2 m/s). This result suggests that birds flew with nearly constant effort in both seasons.

In order to better assess the statistical significance of the seasonal differences in airspeed, we computed the probability that a spring airspeed is higher than autumn airspeed using the exact empirical probability distribution function (Figure 1). Both distributions largely overlapped and the probability that birds fly faster in spring was similar to the probability in autumn (54% of birds flew faster in spring than in autumn and 46% flew faster in autumn).

### 3.2 Daily and intraseasonal scale

**FIGURE 2:**
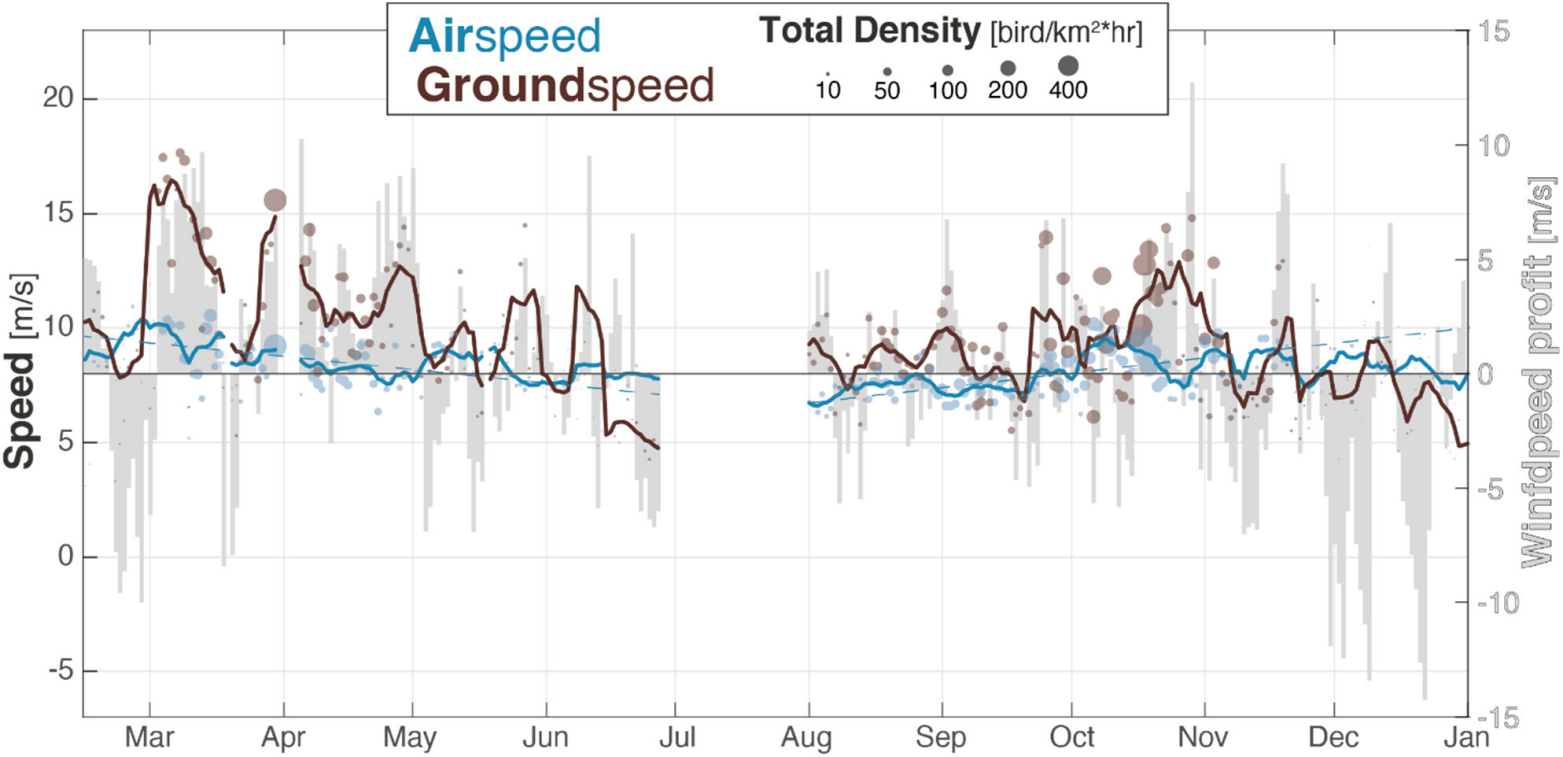
Daily ground speed (brown), airspeed (blue), and wind profit (background gray). Circles indicate the daily average across all radars, and the solid lines represent their 7‐day moving‐average. The size of the circle is proportional to the total number of birds in the air. All averages are weighted by the number of birds (i.e., density). Wind profit is also averaged for all radar nights and is shown on a second right *y*‐axis with an offset of 8 m/s (airspeed average) because of the sign change. Note that both *y*‐axes have the same scale allowing ease of comparison. The fine dotted blue lines represent the linear trend of airspeed for both seasons separately.

When looking at the daily scale (Figure 2), we find that more birds selected nights with positive wind profit and migrated faster when doing so. Bird ground speed and total density both followed the daily variation of wind profit. However, the highest bird densities were not always attained when there was maximal wind profit, but rather when wind profit became positive after a period of negative wind profit (e.g., early April or end October in Figure 2). More importantly, the daily airspeed was less variable than ground speed (airspeed SD = 3.1 m/s; ground speed SD = 5.1 m/s), suggesting that birds generally flew with constant airspeed independently of wind conditions.

Within a migratory season, ground speed showed a strong decrease in spring of −2 m/s per month (95% CI: −2.6 to −1.4 m/s) and a smaller increase in autumn of 0.57 m/s per month (95% CI: 0.19– 0.94 m/s). In comparison, airspeed had a similar rate of −0.53 m/s per month in spring (95% CI: −0.34 to −0.73 m/s) and −0.58 m/s per month in autumn (95% CI: −0.44 to −0.72 m/s). This indicates that after accounting for wind conditions, the change in airspeed was much stronger within a season than between seasons.

### 3.3 Spatial scale

**FIGURE 3:**
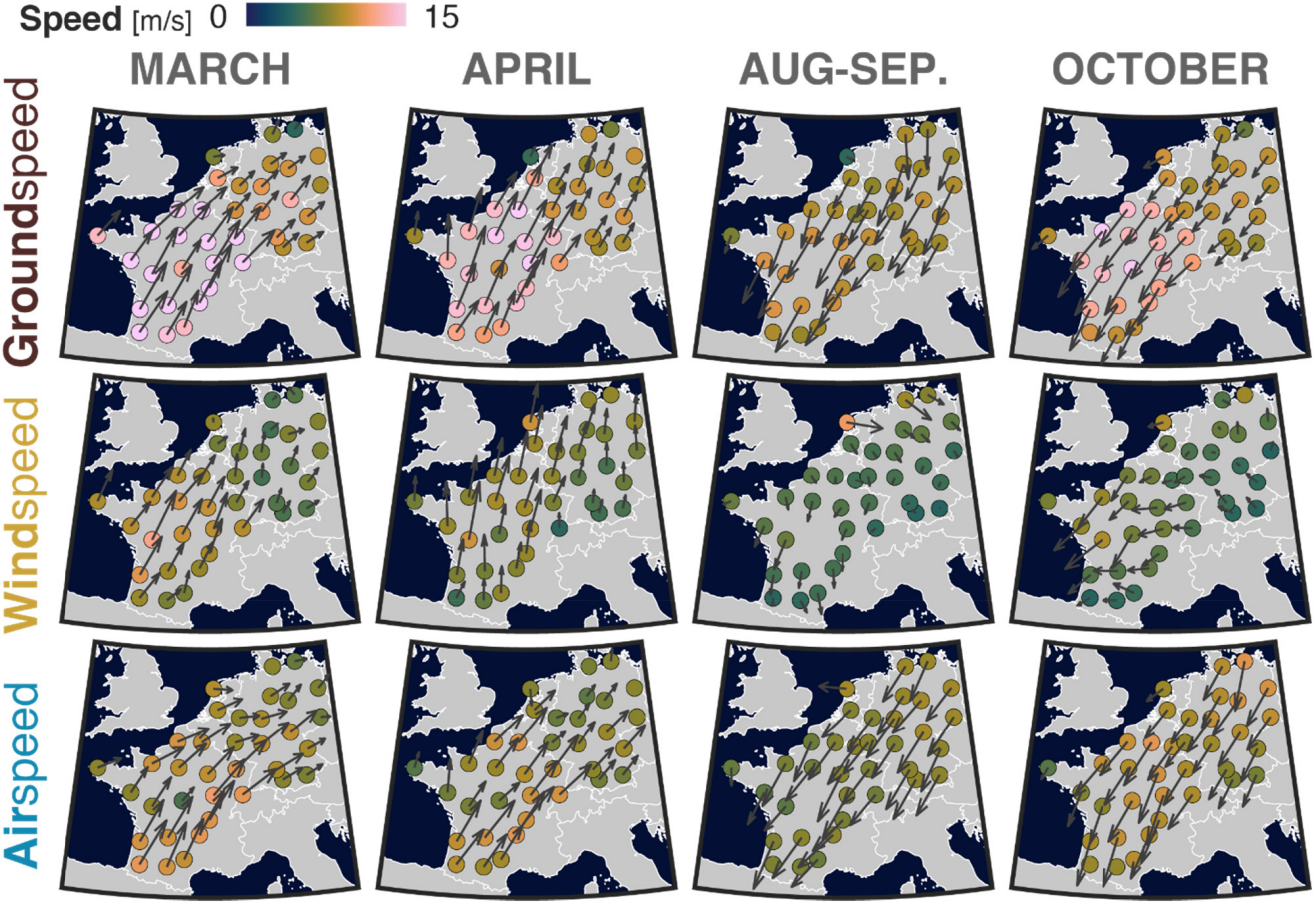
Ground, wind, and airspeed vectorial average by radar weighted by bird density for 4 periods of the year. Both arrow length and circle color indicate speed.

Wind speed was stronger in south‐west Europe than in north‐east Europe (Figure 3), particularly in spring and most strongly in March (compared to the 2000–2019 average in Figure SI‐5). These favorable wind conditions allowed birds to migrate with higher ground speed in this area. More importantly, when removing the wind component from the ground speed, the resulting airspeed showed an impressively uniform spatial pattern for all seasons (SD = 1.03 m/s) compared to wind speed (SD = 1.35 m/s) and ground speed (SD = 2.44 m/s).

### 3.4 Altitudinal scale

**FIGURE 4:**
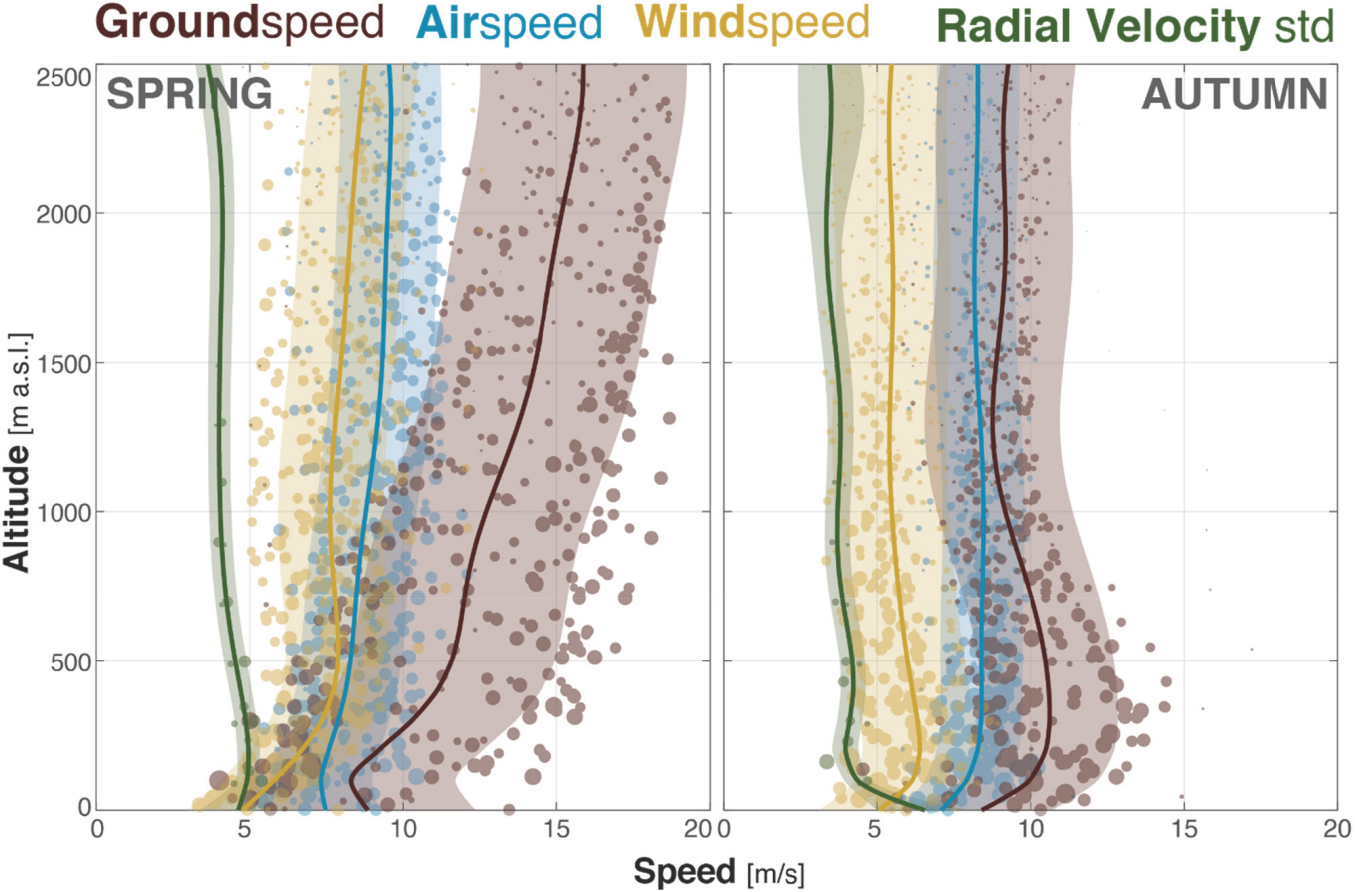
Ground (brown), air (blue), and wind (yellow) speeds and radial velocity standard deviation (green) profiles over altitude for spring (left) and autumn (right). Circles represent the average per radar and altitude bin with the radius proportional to the number of birds (density).

The vertical profile of ground speed resembled the profile of wind speed in both spring and autumn (Figure 4). As a result, the airspeed vertical profile was relatively straight in comparison to ground and wind speed. This indicates that the difference in ground speed with elevation was mainly driven by differences in wind speed, showing there was a relatively constant airspeed irrespective of altitude.

The slight decrease in the standard deviation radial velocity with altitude indicates a more directional flow of birds at higher altitude (i.e., less variance in ground speed). In autumn, the higher standard deviation radial velocity in the first 100 m above ground suggests a high scatter of flight directions, which in turn explains the drop in airspeed (computed as the vectorial average of all birds within radar scanning distance).

## 4 DISCUSSION

We quantified wind assistance for nocturnal mass movements of migratory birds using a large data set covering western Europe with 37 weather radars over a full year. Airspeed is nearly constant across seasons, geography, and altitudes, but wind profit varied between seasons, geography, and altitudes, and consequently, birds migrate faster in spring than in autumn, in France compared to Germany, and at higher altitudes compared to lower altitudes. As flight costs are proportional to airspeed (Hedenström, 2012), our results suggest that nocturnal migrants keep their effort relatively constant across seasons.

### 4.1 The importance of wind in migration speed

Wind speed is an essential factor contributing to birds' overall movement. In this study, birds encountered a wind speed of 6.2 m/s on average (SD: 3.7), while the airspeed was only about 24% higher (8.4 m/s, SD: 3.0). Harnessing the wind, bird ground speed was on average 25% higher than their airspeed (10.5 m/s; SD:5.0; see Figure SI‐1). Given the magnitude of wind speed relative to airspeed, it is crucial for birds to consider wind by minimizing headwind and crosswind while maximizing tailwind (Liechti & McGuire, 2018).

Although wind speed may be high, it does not directly translate into wind profit because the direction of wind is rarely perfectly aligned with the preferred migration direction, requiring birds to compensate for different wind directions. This explains why the average wind profit was only 2.0 m/s (SD: 5.1 m/s; see Figure SI‐2). Our results confirm that birds strategically select the few nights where wind conditions are most favorable (strong wind aligned with preferred direction of migration), during which bird density peaked at a wind profit of 5 m/s (see Figure SI‐2). During those nights, wind profit reached up to 50% of ground speed (see Figure SI‐4).

Our analyses confirm the importance of wind in speeding‐up overall migration by 19% (2.0/10.5). For an average bird migrating a distance of 3000 km in 80 h (assuming a ground speed of 10.5 m/s), wind profit saves 15 h of flight time (or 2–3 nights). As wind profit lowers the energy required to cover a certain distance, it reduces the number of stopovers and/or refueling time and ultimately migration duration.

### 4.2 Spring vs autumn

Based on flight optimization theory, birds should reduce their airspeed with tailwinds and increase airspeed with head‐ or crosswinds in order to minimize flight costs per distance flown (Hedenstrom & Alerstam, 1995; Liechti et al., 1994; Pennycuick, 1978). Lower airspeeds would therefore be expected in spring due to the stronger tail winds.

We observed a 1.3 times higher average ground speed in spring than in autumn (12.6 vs 9.7 m/s), mainly explained by wind conditions in both seasons: wind profit was on average 4.6 m/s in spring and only 1.1 m/s in autumn (see Figure SI‐3) while airspeeds were virtually the same (spring 8.7 vs autumn 8.2 m/s). Thus, wind alone increased overall migration speed by 37% (4.6/12.6) in spring and 11% (1.1/9.7) in autumn. By contrast, the 6% increase in airspeed (8.7/8.2) comes with longer stopovers to refuel, such that the overall increase in migration speed is in fact lower than 6%.

If we assume birds fly based on optimizing time in spring and energy in autumn, the difference in airspeed is expected to be 5–15% (Alerstam, 2003; Nilsson et al., 2013). Although the increase of airspeed we found (5%) falls within this range, it is too small to be considered significant due to the large variance, measurement uncertainty, and data quality as detailed below.

First, weather radars estimate bird ground speed based on the Doppler shift representing the mean vectorial average of all targets (Dokter et al., 2011). Therefore, the ground speed estimated will always be lower than the speed of each individual bird and, more importantly for our study, will decrease as flight directions are more scattered. The alignment of flight directions depends on the variability of the direction followed by each population as well as on the amount of head‐ or tailwind (Bäckman & Alerstam, 2003; Liechti & Bruderer, 1986). The degree of directionality can be assessed with the value of the standard deviation of the radial velocity. We found a higher standard deviation of the radial velocity in spring than in autumn (see Figure SI‐6), which is in line with the results of a bird tracking radar study (Shi et al., 2021). Therefore, the slightly lower autumn airspeed estimated in this study could be explained by birds migrating in more diverse directions. This could be caused by the presence of more unexperienced birds (juveniles) in autumn, whose preferred orientation tends to be more scattered (Åkesson et al., 2021).

Second, the presence of insects with their lower airspeeds reduces the ground speed estimated by weather radars. As insects are more common in autumn than in spring, the average airspeed will be lower in autumn than in spring. Following Nussbaumer et al. (2021), the insect‐to‐bird ratio is modeled based on airspeed and standard deviation radial velocity, accounting for both time and space variation. The ground speed of birds was then corrected based on the estimated insect‐to‐bird ratio and the fitted distribution of birds' and insects' airspeed. While this approach is currently the best available (e.g., compared to strict airspeed thresholds), the ground speed correction is not perfect, and therefore a slight residual influence of insect contamination in the autumn data cannot be excluded.

We compare the spring/autumn speed ratios with other studies using different radar techniques, spatial and temporal coverage see Table SI‐1. In general, radars tracking single targets are more reliable in estimating the exact speed of individual birds. However, tracking radars are more prone to bias because (i) closer and larger birds are more likely to be tracked and (ii) they can only track a single bird at the time, tracking relatively fewer birds during high than during low migration intensity, the latter often being associated with less favorable winds. Of all the radar data sets, military tracking radars are generally considered the most reliable to estimate airspeed (Nilsson et al., 2018).

Most tracking radar studies have shown that airspeeds are not significantly higher in spring than in autumn (Bäckman & Alerstam, 2003; Kemp et al., 2010; Liechti & Bruderer, 1995), while other studies found significantly higher airspeeds in spring (Green & Alerstam, 2000; Karlsson et al., 2012). Interestingly, the tracks used in studies by Bäckman and Alerstam (2003) and Karlsson et al. (2012) were collected with the same radar at the same site. However, while Karlsson et al. (2012) focused on early autumn (August) and late spring (May), Bäckman and Alerstam (2003) collected data during late autumn (October) and early spring (April), with hardly any overlap between the observation periods. If we restrict our dataset to the same periods as Karlsson et al. (2012), we obtain a 1.11 times faster airspeed in spring (8.3 m/s) than in autumn (7.5 m/s).

In the northeast of the United States, Horton, van Doren, Stepanian, Farnsworth, and Kelly (2016a) found a ratio of 1.23 times faster airspeed in spring than autumn for six weather radars. We can only speculate that this difference is at least partially linked to the larger scattering of flight directions, or to their treatment of insect contamination. Surprisingly, they excluded about the same amount of insect contamination in spring and autumn, although we would expect more insects in autumn (Larkin, 1991; Nussbaumer et al., 2021; Shi et al., 2021). In addition, with half of their radars located on the coast, one can also expect that the strong seasonally dependant coastal effect (Horton, van Doren, Stepanian, Hochachka, et al., 2016) might cause increased airspeed in autumn (e.g., more compensation required with wind blowing bird offshore).

While these earlier studies are either geographically limited, prone to sampling biases or do not cover the entire year, our results comprehensively analyze the spatio‐temporal differences in ground, air, and wind speed to highlight the absence of significant increase of airspeed in spring compared to autumn.

### 4.3 Early vs late migration (long‐distance vs short‐distance)

Our analysis shows a decrease in airspeed during spring migration and an increase during autumn. This shift in airspeed could be associated with a gradual change in the species composition in autumn from small trans‐Saharan migrants to medium‐sized short‐distance migrants, and vice versa, in spring. This result is consistent with previous tracking radar studies (Dokter et al., 2011; Liechti, 1992; Nilsson et al., 2014) and a citizen science‐based study (Horton et al., 2018). In addition, spatial variation in species composition could also cause the small spatial difference in airspeed observed (see Figure 3 and Figure SI‐7). Optimal flight theory predicts a maximum range airspeed of 7.4 m/s for a trans‐Sahara migrant such as a Willow Warbler and a 12 m/s airspeed for a short‐distance migrant such as a Song Thrush (Pennycuick, 2008).

### 4.4 Altitude effect

In general, wind speed increases with altitude and in the northern hemisphere wind directions tends to turn clockwise (North et al., 2014). Therefore, by choosing a specific flight altitude, a bird can select specific wind conditions (Shamoun‐Baranes et al., 2017). Birds tend to fly in the first kilometers of the atmosphere in temperate zones (Bruderer et al., 2018; la Sorte et al., 2018), but some birds have been observed flying at extremely high altitude to benefit from high wind support (Liechti & Schaller, 1999; Senner et al., 2018).

There is a general consensus that birds fly at the first altitude with favorable wind (Bruderer et al., 1995; Bruderer & Liechti, 1995; Dokter et al., 2013; Horton, van Doren, Stepanian, Farnsworth, & Kelly, 2016b; Kemp et al., 2013; Mateos‐Rodríguez & Liechti, 2012) irrespective of temperature and humidity conditions (Liechti & Schmaljohann, 2007; Schmaljohann et al., 2009). Thus, with supporting winds at higher altitude, spring migration generally occurs at higher altitude than autumn migration (Dokter et al., 2013; Horton, van Doren, Stepanian, Farnsworth, & Kelly, 2016b; Shamoun‐Baranes et al., 2017) but see (la Sorte et al., 2015).

Because air density decreases with altitude, optimal theory predicts an increase in airspeed with altitude (Bruderer et al., 2018; Hedenstrom & Alerstam, 1995), which has been supported by empirical results (Bruderer, 1971; Hedenström et al., 2002; Schmaljohann & Liechti, 2009). Our results reveal only a small increase of airspeed with height, with a slightly stronger pattern in spring than in autumn, but the vertical variation in groundspeed is dominated by the seasonal difference in tail winds.

## AUTHOR CONTRIBUTIONS

**Raphaël Nussbaumer:** Conceptualization (equal); data curation (lead); formal analysis (lead); visualization (lead); writing – original draft (lead). **Baptiste Schmid:** Conceptualization (equal); data curation (supporting); formal analysis (supporting); writing – review and editing (equal). **Silke Bauer:** Conceptualization (equal); writing – review and editing (equal). **Felix Liechti:** Conceptualization (equal); data curation (supporting); formal analysis (equal); writing – review and editing (equal).

## FUNDING INFORMATION

This research was funded by the Swiss National Science Foundation (grant no. 191138). This project is part of GloBAM funded through the 2017–2018 Belmont Forum and BiodivERsA joint call for research proposals, under the BiodivScen ERA‐Net COFUND programme, and with the funding organizations Swiss National Science Foundation (SNF 31BD30_184120), Belgian Federal Science Policy Office (BelSPO BR/185/A1/GloBAM‐BE), Netherlands Organization for Scientific Research (NWO E10008), Academy of Finland (aka 326315) and National Science Foundation (NSF 1927743, NSF 2017817).

## CONFLICT OF INTEREST

The authors have no conflicts of interest to declare that are relevant to the content of this article.

## Supporting information


Appendix S1

## Data Availability

The weather radar data used for this study are available at https://doi.org/10.5281/zenodo.3610184 (Nussbaumer, 2020). The MATLAB livescript used to perform the analysis and generate the figures is included in the Supporting Information and the full project repository is accessible at https://github.com/Rafnuss‐PostDoc/BMM/tree/master/WindSupport.
