# Supplementary material for: Favorable winds speed up bird migration in spring but not in autumn
Source: Ecol Evol. 2022 Jul 31;12(8):e9146. doi: 10.1002/ece3.9146 (PMC9339755; doi:10.1002/ece3.9146)
Supplement: Supplementary file 1 — Appendix S1 [file ECE3-12-e9146-s001.docx]

Supporting Information


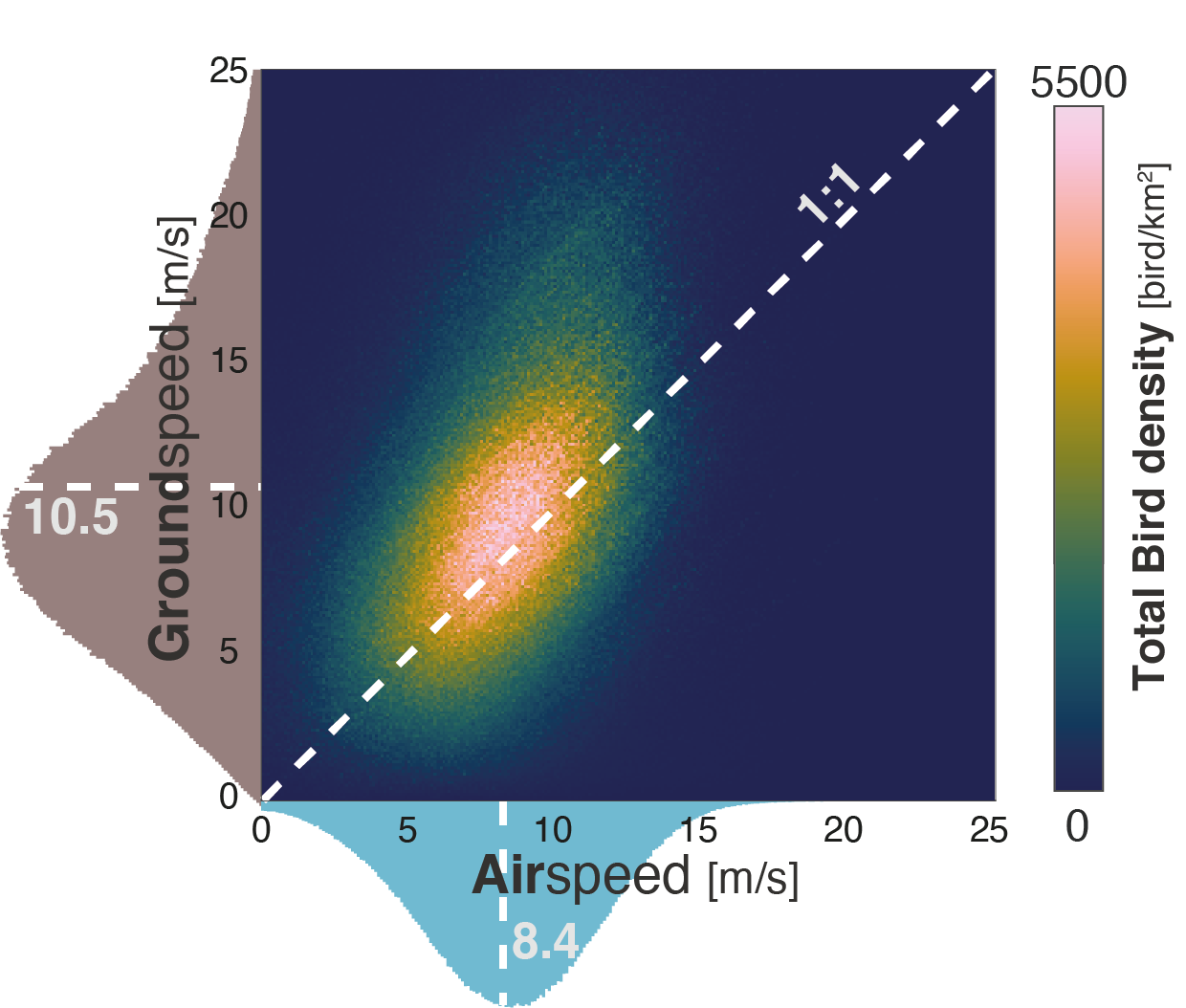


Figure SI-1: 2D histogram of all datapoints (radar-timestep) for groundspeed and airspeed weighted by bird density (bins size of 0.1 m/s) The background axis represents the histogram of the airspeed and groundspeed separately (also weighted by bird density), together with their respective mean. Most bird migrate with a higher groundspeed than airspeed (above the 1:1 line).


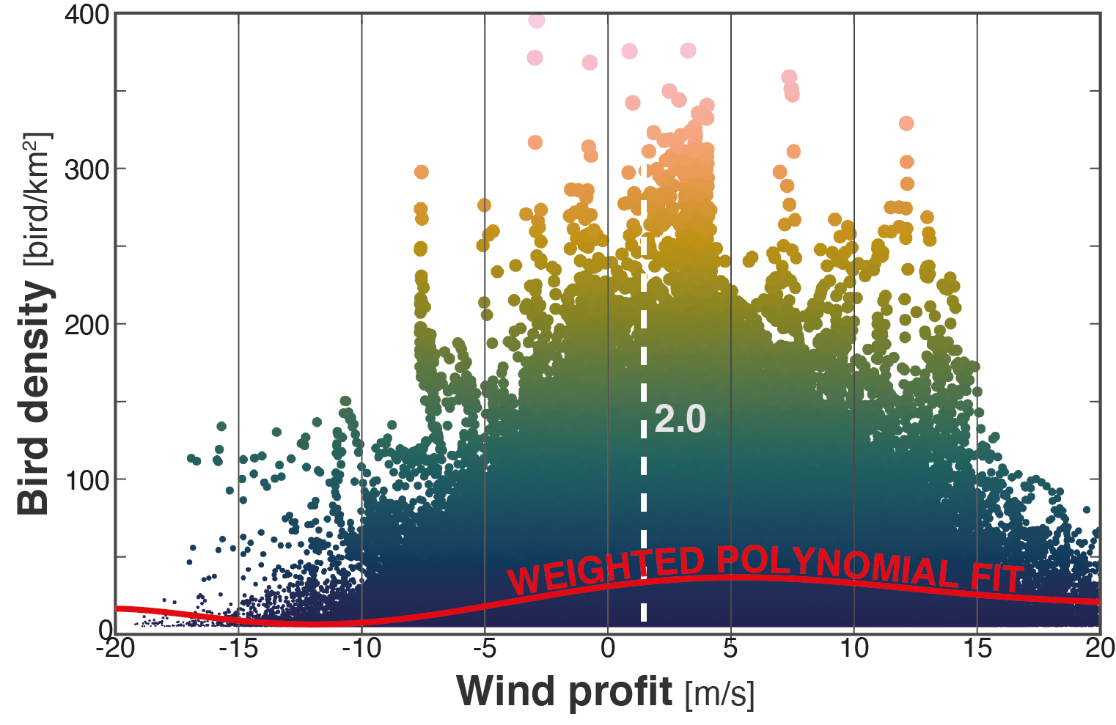


Figure SI-2: Bird density generally increases with positive wind profit, peaking around 5m/s, but with a weighted average of only 2.0m/s.


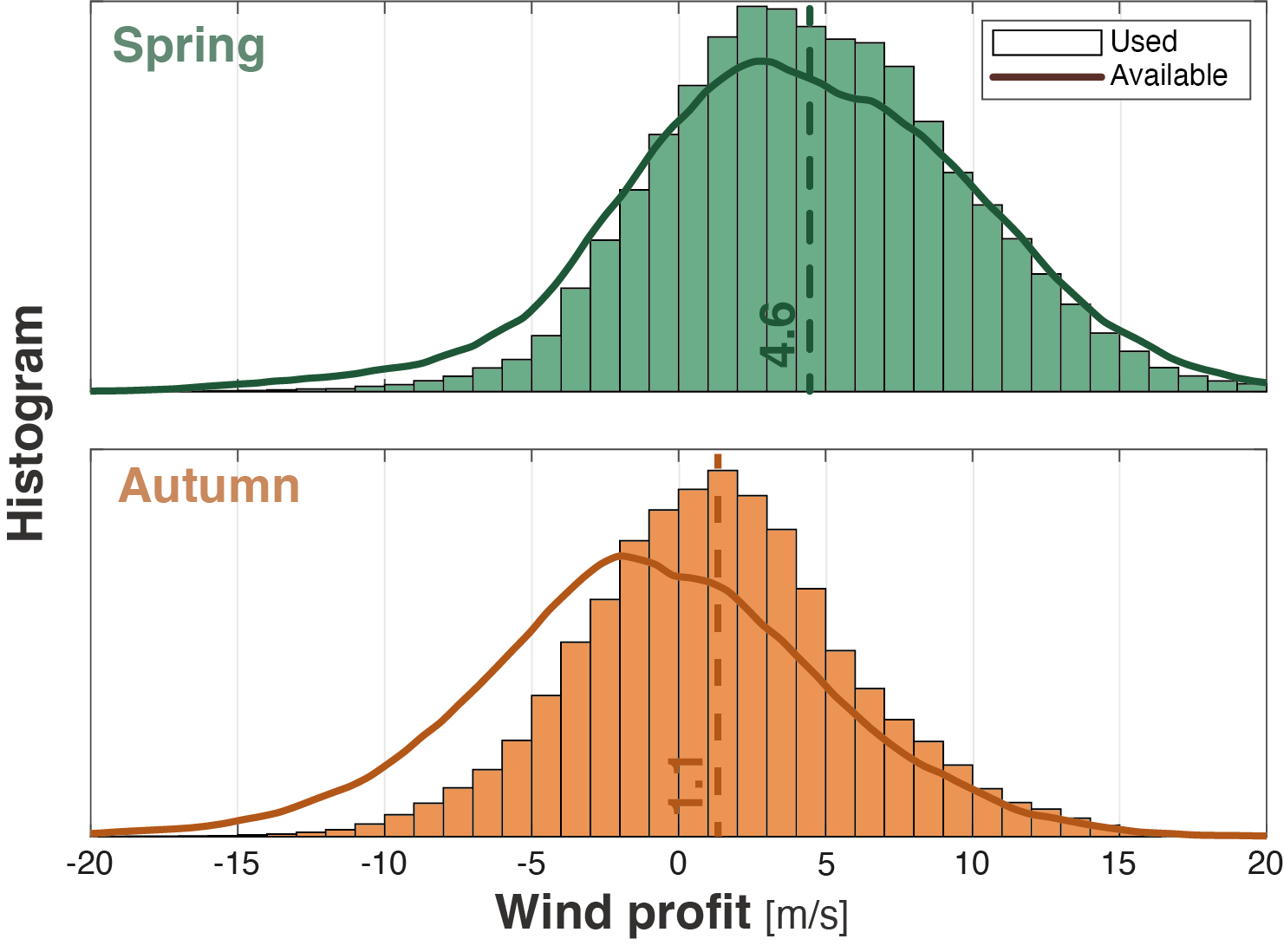


Figure SI-3: Histogram of wind profit weighted by bird density for used and not weighted for available.


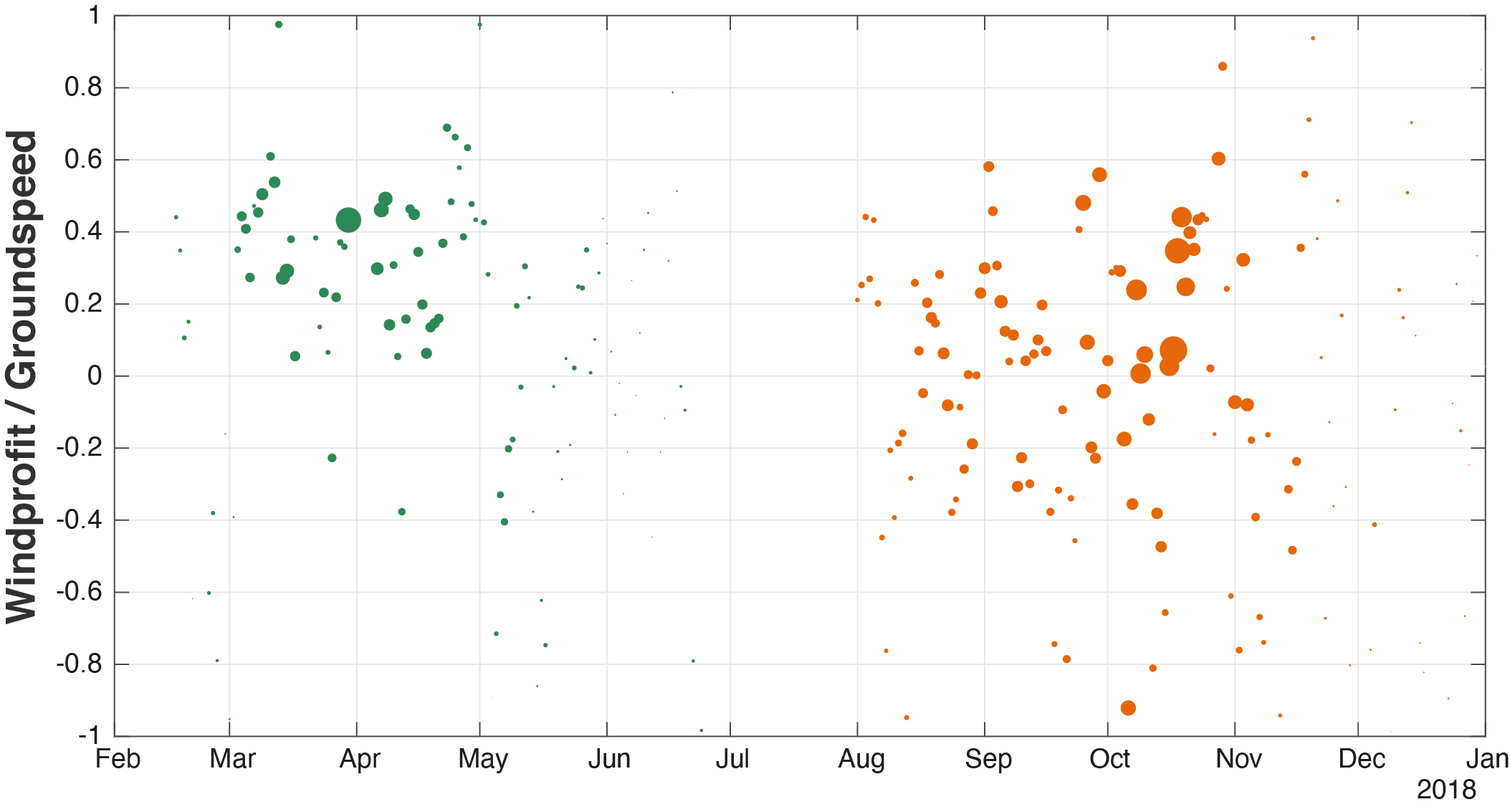


Figure SI-4: Average ratio of the displacement due to wind (windprofit/groundspeed). Size is proportional to total density.


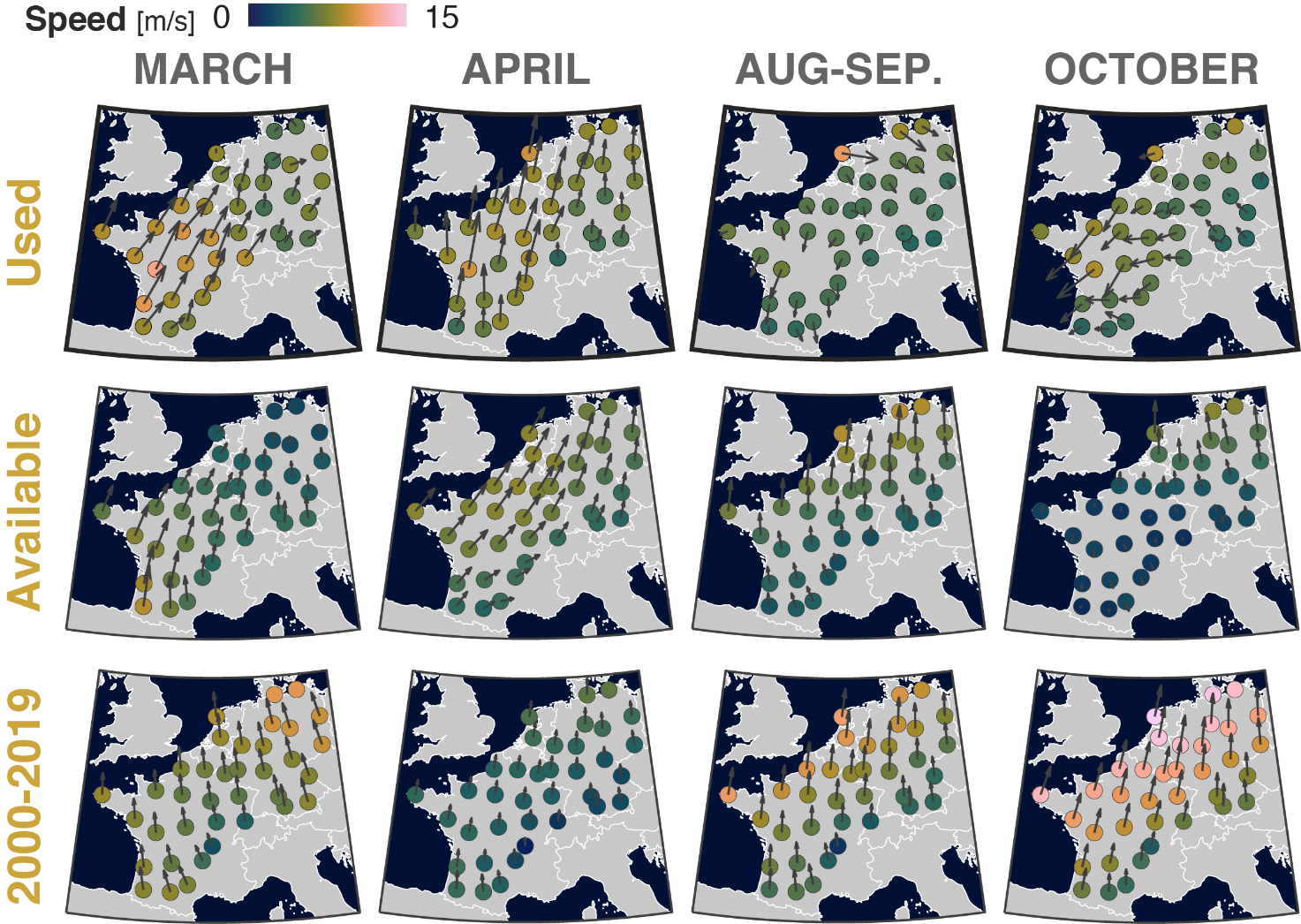


Figure SI-5: Comparison of the spatial distribution of wind speed between used, available (in 2018) and the average 2000-2019.


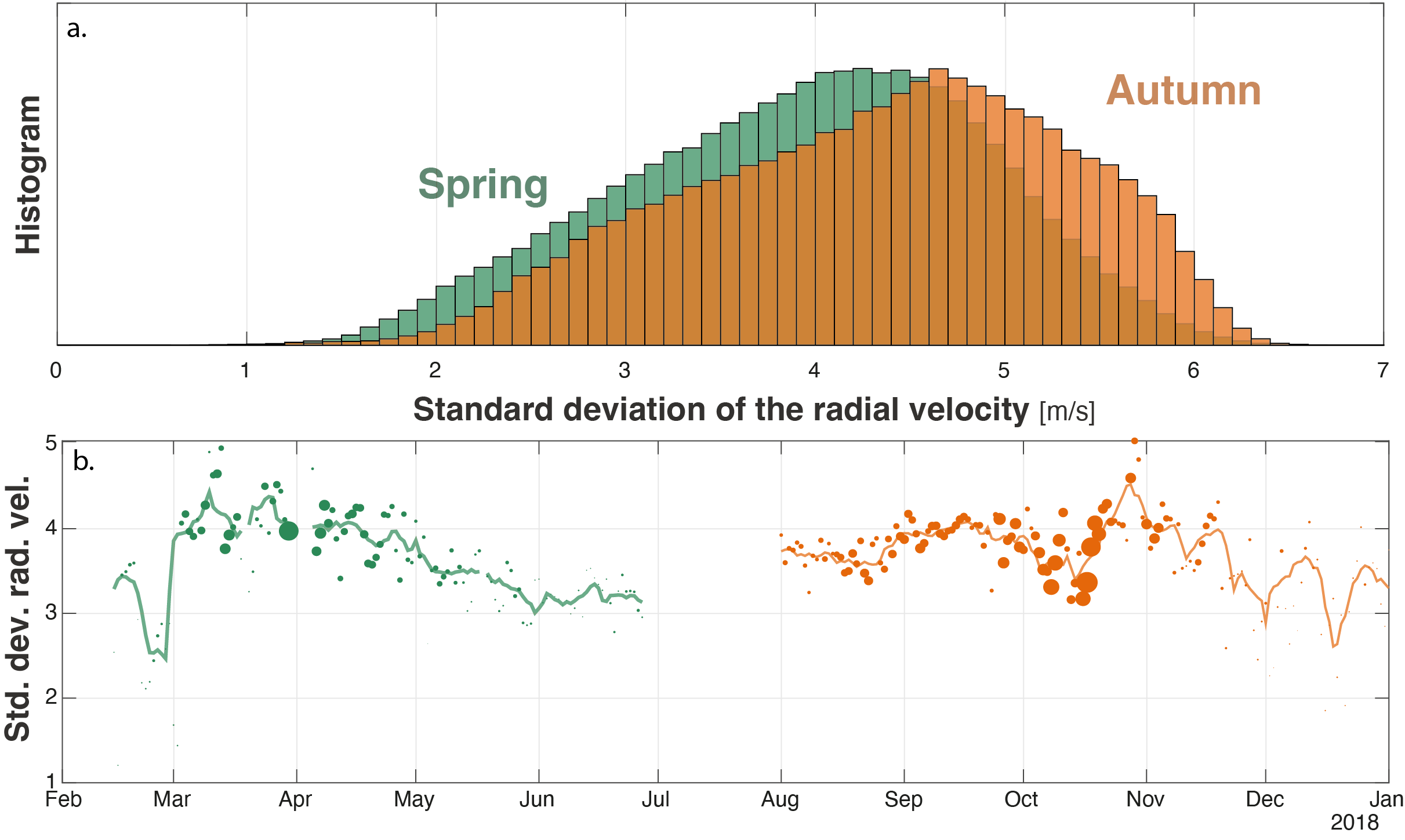


Figure SI-6: Standard deviation of radial velocity measured by the weather radar (a) in spring and autumn and (b) each day of the year with moving average of 7 days.

Figure SI-7. Spring/autumn ratios for each weather radar (color corresponds to latitude)

Table SI-1: Comparison of spring vs autumn

|  | Technique | Spatial coverage | Temporal coverage | SamplE size | Groundspeed | | | | Airspeed | | |
| --- | --- | --- | --- | --- | --- | --- | --- | --- | --- | --- | --- |
|  |  |  |  |  | Spring | Autumn | | Ratio | Spring | Autumn | Ratio |
| This study | Weather radar (C-band) | FR, GE, NL, BE | Full year 2018 | 37 WR | 12.6 (5.8) | | 9.7 (4.5) | 1.3 | 8.7 (3.3) | 8.2 (2.9) | 1.06 |
| (Horton, Van Doren, Stepanian, Farnsworth, et al. 2016a) | Weather radar (S-band) | northeast USA | Spring 2013- Spring 2015 | 6 WR | 14.6 | 10.6 | | 1.38 | 10.6 | 8.3 | 1.23 |
| (unpublished) | Birdscan | Sempach (CH) | 2018 | 88’606 | 14.8 (7.5) | 13.6 (6.1) | | 1.06 | 13.9 (6.9) | 13.5 (6.1) | 1.03 |
| (Kemp et al. 2010) | ﻿long-range medium-power stacked-beam radar | Wier (NL) | Spring 2006- Autumn 2008 | 6’701’202 | 22.1 (5.5) | 18.9 (3.7) | | 1.17 | 13.6 (3.6) | 14.0 (4.7) | 0.97 |
| (Karlsson et al. 2012) | Tracking (X-band) | Lund (SW) | 2004-2008 | 3’088 | 12.7 (4.2) | 10.5 (4.1) | | 1.21 | 11.9 (2.4) | 10.5 (2.3) | 1.13 |
|  |  | Abisko (SW) | 2004-2008 | 3’088 | 11.3 (4.9) | 10.2 (3.3) | | 1.11 | 11.5 (2.5) | 9.5 (2.1) | 1.21 |
|  | Tracking (X-band) | Lund (SW) | ﻿1999 | 1’297 |  |  | |  |  |  |  |
| (Liechti and Bruderer 1995) | Tracking | Arava (IS) | 1991/92 | 15’451 | 12.7 (4.4) | 14.1 (3.6) | | 0.905 | 11.8 (3) | 11.5 (2.8) | 1.03 |
|  |  | Mallorca (ES) | 1996/97 | 13’746 | 13.1 (5.1) | 13.4 (4.6) | | 0.978 | 11.3 (3.2) | 11.4 (3) | 0.99 |
|  |  | Ouadane (MO) | 2003/04 | 18’907 | 14.1 (5.3) | 13.4 (5.1) | | 1.05 | 11.8 (3.4) | 11.7 (3.5) | 1.01 |
|  |  | Fehmarn (GE) | 2009/10 | 2’185 | 14.6 (5.1) | 14.5 (4.8) | | 1.01 | 13.9 (3.6) | 13.9 (3.6) | 1.00 |
|  |  | Malaga (ES) | 1996/97 | 22’592 | 11.8 (4.3) | 11.4 (4) | | 1.04 | 11.5 (3.4) | 11.3 (3.4) | 1.01 |
|  |  | Sede Boqer (IS) | 1991/92 | 9’878 | 14 (4.4) | 14.7 (3.7) | | 0.952 | 12.1 (3) | 11.8 (2.9) | 1.03 |
|  |  |  |  | 83’085 | 13.1 (5.2) | 12.3 (4.5) | | 1.07 | 11.9 (3.4) | 11.6 (3.2) | 1.03 |
